# Harold Bourne, MB BS, DPM, FRANZCP, FRCPsych

**DOI:** 10.1192/bjb.2019.19

**Published:** 2019-08

**Authors:** Brian Barraclough

Formerly Senior Lecturer in Psychiatry, Otago University, Dunedin, New Zealand; Consultant Child and Adolescent Psychiatrist, Charing Cross Hospital, London, UK

**Figure:**
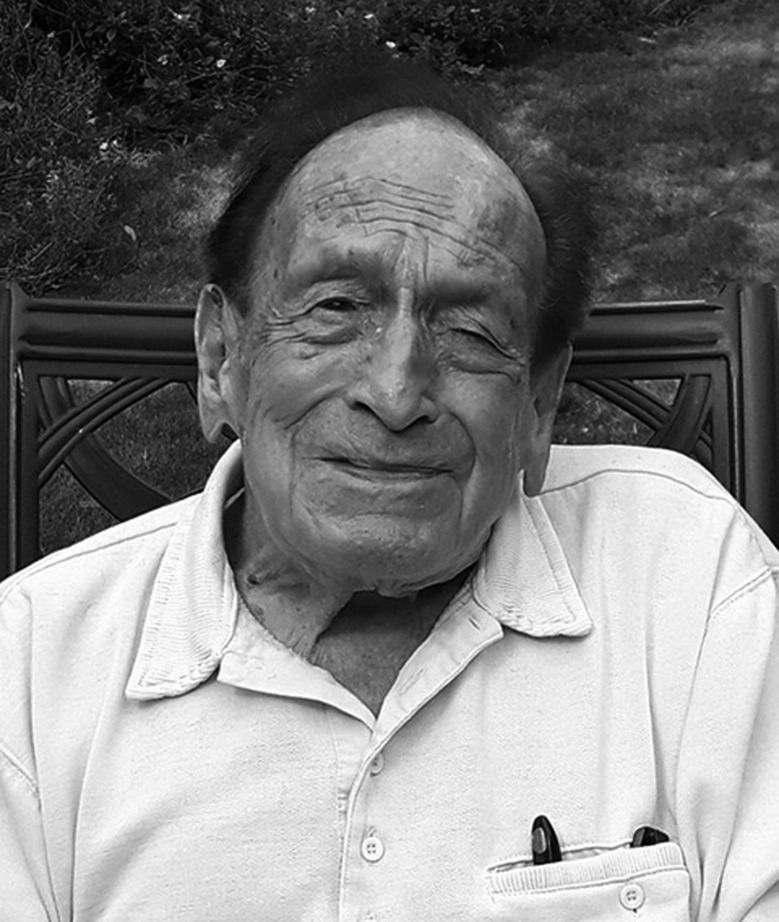
Harold Bourne at 94

Those who pioneer new treatments in psychiatry are usually held in high esteem. Those who successfully question established but potentially harmful interventions, though equally important, are often forgotten. This has been the case with Harold Bourne, who died recently at the age of 95 in London. In the early 1950s, while working at Netherne Hospital (a 2000 bed mental hospital in Surrey), Bourne began to question the use of insulin coma in the treatment of early schizophrenia. He wrote a notable paper, ‘The Insulin Myth’,^1^ published in *The Lancet* in 1953. It was a review of the evidence for the effectiveness of insulin coma for schizophrenia and it concluded that the treatment was ineffective. Insulin coma, sometimes enhanced with electroconvulsive therapy during the coma, was the standard treatment for schizophrenia from the early 1930s until the late 1950s and all mental hospitals had insulin units to administer it. For a 30-year-old junior doctor this was a courageous act which challenged the authority of the profession's leaders. In 1957 (4 years after the publication of Bourne's paper), Brian Ackner and colleagues published the results of a controlled trial showing there was no difference in outcome between barbiturate-induced coma and insulin coma.^2^ Shortly afterwards, all insulin units began to shut and this dangerous treatment went out of use.

Two other papers deserve mention. Bourne's paper on convulsion dependence^3^ described persistent psychoses which remained in remission only with long-term electroconvulsive therapy. This was before depot phenothiazines became available. Further, from his experience at the 600-bed Fountain Hospital for the severely mentally handicapped came his neologism ‘protophrenia’, a term for the dwarfing effect on mental development in children due to socially impoverished child-rearing, which could be mistaken for mental handicap caused by neuropathology.^4^ This latter observation preceded the description of psychosocial short stature or deprivation dwarfism by some 15 years.

Harold was the elder son of Jack Baum and Rachel, née Oster, and born in 1923 into a traditional London East End Ashkenazi Jewish family. His father was a tailor from Poland; his mother a school teacher. She was born in London, the daughter of Jewish migrants from Eastern Europe. Her brother was a family doctor which may have influenced Harold in his choice of career. The family anglicised their name to Bourne in 1934 but Harold always remained proud of his Jewish heritage.

Harold, known as ‘Boy’ in his family, won a scholarship to a grammar school in Islington, Dame Alice Owen's School. He then read medicine at University College London. His life-long interest in psychoanalysis which had begun at school was developed through his friendship with Charles Rycroft (1914–98), a contemporary at University College Hospital. Rycroft, 10 years older, was then completing his own analysis while studying medicine.

Following qualification, after 2 years of National Service at a 400-bed military psychiatric unit at Banstead Hospital and posts at Netherne and the Fountain Hospital, in 1955 Bourne was appointed Lecturer in Psychiatry in the Faculty of Medicine at the University of Otago, ultimately becoming Senior Lecturer and Senior Psychiatric Physician at Dunedin Hospital. The move to Dunedin, so he said, was to distance himself and his family from the European nuclear war he saw as imminent.

Bourne was a breath of fresh air in Dunedin, then an inward-looking academic community isolated by distance from its parent culture. He introduced the ideas of Freud and the belief that psychoanalysis could explain the causes of mental disorder and provide a treatment for them. In the absence of staff to provide this specialised treatment he used electroconvulsive therapy and drugs.

Bourne's charm, intellect and capacity for friendship influenced students and junior doctors to consider psychiatry, which was then a neglected and stigmatised specialty. He was an inspiring teacher of psychiatry with many protégées. Among those he influenced and who had careers in psychiatry in England were John Denford, Margaret Rich, John Steiner and myself. At least ten others had careers in Australia and North America.

In 1974 Bourne returned to London to a National Health Service child and adolescent psychiatry post at Charing Cross Hospital. He also established a therapeutic community at St Bernard's Hospital.

On retirement at 65 years of age in 1988 he became a psychotherapist to the expatriate anglophone community in Rome. Infirmity compelled a return to England in 2014 where he lived in a Jewish old people's residential home in Golders Green until he died.

Bourne married three times. In 1945 he married Winifred Hickson, known as Freddie. They divorced in 1980. In 1981 he married Niloufer Hickman-Fitter, divorcing in 1988. In 1989 he married Flavia Donati; they separated in 1998. With Freddie he had five children, with Niloufer one and with Flavia two. He is survived by Niloufer, Flavia, eight children, nine grandchildren, two great-grandchildren and his brother Stanford (known as Sandy) – a London psychoanalyst.

Harold Bourne was born in London 9 April 1923 and died in London 6 November 2018.

## References

[1] Bourne H. The insulin myth. Lancet 1953; 265: 964–8. 13110026 10.1016/s0140-6736(53)90622-9

[2] Ackner B, Harris A, Oldham AJ. Insulin treatment of schizophrenia; a controlled study. Lancet 1957; 272: 607–11. 13407078 10.1016/s0140-6736(57)91070-x

[3] Bourne H. Convulsion dependence. Lancet 1954; 267: 1193–6. 13213158 10.1016/s0140-6736(54)92259-x

[4] Bourne H. Protophrenia: a study of perverted rearing and mental dwarfism. Lancet 1955; 267: 247. 10.1016/s0140-6736(55)92895-6 13272348

